# Risk for transfusion-transmitted infectious diseases in Central and South America.

**DOI:** 10.3201/eid0401.980102

**Published:** 1998

**Authors:** G A Schmunis, F Zicker, F Pinheiro, D Brandling-Bennett

**Affiliations:** Pan American Health Organization, Washington, D.C., USA. schmunig@paho.org

## Abstract

We report the potential risk for an infectious disease through tainted transfusion in 10 countries of South and Central America in 1993 and in two countries of South America in 1994, as well as the cost of reagents as partial estimation of screening costs. Of the 12 countries included in the study, nine screened all donors for HIV; three screened all donors for hepatitis B virus (HBV); two screened all donors for Trypanosoma cruzi; none screened all donors for hepatitis C virus (HCV); and six screened some donors for syphilis. Estimates of the risk of acquiring HIV through blood transfusion were much lower than for acquiring HBV, HCV, or T. cruzi because of significantly higher screening and lower prevalence.rates for HIV. An index of infectious disease spread through blood transfusion was calculated for each country. The highest value was obtained for Bolivia (233 infections per 10,000 transfusions); in five other countries, it was 68 to 103 infections per 10,000. The risks were lower in Honduras (nine per 10,000), Ecuador (16 per 10,000), and Paraguay (19 per 10,000). While the real number of potentially infected units or infected persons is probably lower than our estimates because of false positives and already infected recipients, the data reinforce the need for an information system to assess the level of screening for infectious diseases in the blood supply. Since this information was collected, Chile, Colombia, Costa Rica, and Venezuela have made HCV screening mandatory; serologic testing for HCV has increased in those countries, as well as in El Salvador and Honduras. T. cruzi screening is now mandatory in Colombia, and the percentage of screened donors increased not only in Colombia, but also in Ecuador, El Salvador, and Paraguay. Laws to regulate blood transfusion practices have been enacted in Bolivia, Guatemala, and Peru. However, donor screening still needs to improve for one or more diseases in most countries.


### Risk for Transfusion-Transmitted Infectious Diseases in Central and South America

**Gabriel A. Schmunis, Fabio Zicker, Francisco Pinheiro, and David Brandling-Bennett**
Pan American Health Organization, Washington, D.C., USA

We report the potential risk for an infectious disease through tainted transfusion in 10 countries of South and Central America in 1993 and in two countries of South America in 1994, as well as the cost of reagents as partial estimation of screening costs. Of the 12 countries included in the study, nine screened all donors for HIV; three screened all donors for hepatitis B virus (HBV); two screened all donors for *Trypanosoma cruzi;* none screened all donors for hepatitis C virus (HCV); and six screened some donors for syphilis. Estimates of the risk of acquiring HIV through blood transfusion were much lower than for acquiring HBV, HCV, or *T. cruzi* because of significantly higher screening and lower prevalence rates for HIV. An index of infectious disease spread through blood transfusion was calculated for each country. The highest value was obtained for Bolivia (233 infections per 10,000 transfusions); in five other countries, it was 68 to 103 infections per 10,000. The risks were lower in Honduras (nine per 10,000), Ecuador (16 per 10,000), and Paraguay (19 per 10,000). While the real number of potentially infected units or infected persons is probably lower than our estimates because of false positives and already infected recipients, the data reinforce the need for an information system to assess the level of screening for infectious diseases in the blood supply. Since this information was collected, Chile, Colombia, Costa Rica, and Venezuela have made HCV screening mandatory; serologic testing for HCV has increased in those countries, as well as in El Salvador and Honduras. *T. cruzi* screening is now mandatory in Colombia, and the percentage of screened donors increased not only in Colombia, but also in Ecuador, El Salvador, and Paraguay. Laws to regulate blood transfusion practices have been enacted in Bolivia, Guatemala, and Peru. However, donor screening still needs to improve for one or more diseases in most countries.

Preventing the transmission of infectious diseases through blood transfusion in developing countries is difficult given that the resources needed are not always available, even when policies and strategies are in place (1). Avoiding paid donors, selecting blood donors through questionnaires, and limiting the number of transfusions can prevent the transmission of infections. Testing for specific antibodies is the final measure for eliminating unsafe blood.

The risk for transfusion-transmitted infectious diseases can be estimated on the basis of screening level for each infectious agent and the prevalence rate of the infection in the donor population. Estimates may also take into account the sensitivity, specificity, and window period of the testing assays. We report here an estimate of such a risk in 12 Central and South American countries and the cost of reagents required for the screening of these infectious diseases as a proxy of resources needed to reduce the risk.

### Source of Information

This report analyzes data from 1993 on screening of blood donors from five countries (Costa Rica, El Salvador, Guatemala, Honduras, and Nicaragua) of Central America. Data were also analyzed for 1993 from five countries

Address for correspondence: Gabriel A. Schmunis, Pan American Health Organization—PAHO, 525 23th Street NW, Washington, D.C. 20037, USA; fax: 202-974-3688; e-mail: schmunig@paho.org.

(Bolivia, Chile, Colombia, Peru, and Venezuela) and for 1994 from two countries (Ecuador and Paraguay) of South America. Information was obtained from Ministry of Health reports during technical meetings in which the situation of each country was reviewed (2-5) or from an official report (6).

In addition, data are presented on the least expensive reagents for detecting antibodies for HIV, hepatitis C virus (HCV), *Trypanosoma cruzi*, and *Treponema pallidum*, and for detecting hepatitis B virus (HBV) antigen (HBsAg) (2,3). All data are national except for Peru, where the information was for the city of Lima only (3). Population data were from the Pan American Health Organization's publication Health Conditions in the Americas (7). Estimates are based on reported results of donor screening activities (2-6).

### Assumptions

For the best possible scenario, the following assumptions were made: 1) Because the laboratory procedures and brands of reagents used in the 12 countries may differ in sensitivity and specificity, comparisons between them are not straightforward. In addition, results of the screening are influenced by the existence of an organized system of quality control and proficiency testing for the serology and for the evaluation of the diagnostic kits, which most countries lacked from 1993 to 1994. Most countries reported the use of different brands of second, third, and fourth generation immunologic assays for screenings of HCV, HIV, and HBV, respectively. Therefore, we assumed that the specificity of the tests for viral diagnosis was 100%, but the sensitivity was 90.00% for HCV, 99.99% for HIV, and 99.90% for HBV. These specificity and sensitivity estimates fit well with those reported for second, third, and fourth generations of assays for HCV, HIV, and HBV, respectively, as mentioned in the package insert by two of the manufacturers of reagents used in the countries. Average window periods for those assays were 20 to 25 days (8,9), 82 to 84 days (9,10), and 51 days (9) for HIV, HBV, and HCV, respectively. In the case of *T. cruzi* serology, we selected the upper range of reported sensitivity and specificity (90% and 95%, respectively) (11,12). For *T. cruzi*, the probability that a person may become a donor during the window period is low because infection is usually acquired in childhood and in rural areas. 2) We assumed that prevalence of infection in unscreened donors was the same as the national average prevalence for each infectious disease. 3) Chile (6) and Peru (3) were the only countries that reported a fractionation index, 1.85 and 1.5, respectively. As no other country provided data on the fractionation index or data allowing one to be calculated, to put the countries in the same category, it was assumed that every blood donation corresponded to a single transfusion to one recipient.

### Screening Coverage and Prevalence Rates

Table 1 shows coverage of screening and prevalence rates of seropositive tests for specific infectious agents among blood donors reported by each of the 12 countries. For HIV, 100% of the donors were screened in all countries, except Bolivia (36.20%), Ecuador (89.50%), and Colombia (98.80%). Prevalence rates for HIV varied from 3.90 per 1,000 in Honduras to 0.04 per 1,000 in Nicaragua. For HBV, only Costa Rica, Peru, and Venezuela screened 100% of donors. The highest values of HBV prevalence estimated were 14.40 per 1,000 for Venezuela and 13.00 per 1,000 for Paraguay. Bolivia, Costa Rica, and Paraguay did not screen for HCV at all, and all other countries screened fewer than 58% of donors; prevalence rates varied from 0.50 to 9.40 per 1,000. Screening for syphilis was not complete in Bolivia, Chile, Colombia, Ecuador, Nicaragua, and Paraguay; prevalence rates were 5.00 to 28.00 per 1,000. For *T. cruzi* infection, only Venezuela and Honduras screened 100% of donors; prevalence rates were 2.00 per 1,000 in Ecuador to 147.90 per 1,000 in Bolivia. In 1993, Peru and Costa Rica had not yet introduced screening for *T. cruzi*.

### Estimating Potential Infectivity of the Blood Supply

The probability of receiving an infected transfusion unit $P(R)$ in each country was estimated by multiplying the prevalence of a specific infection by 1-level of screening (Table 1). For those estimates, the sensitivity and specificity of the different tests were taken into account. As the overall assumed sensitivity of HIV screening was 99.99%, the adjustment of prevalence rates makes no material difference to the precision of the figures in Table 1. The probability of getting a transfusion-transmitted infection $P(I)$ was calculated as the result of the probability of receiving an infected transfusion

Table 1. Coverage of screening[a] of blood donors and seroprevalence rates (per 1,000) of infectious diseases, by country[b]

| | HIV | | HBV[c] | | HCV | | Syphilis | | *T. cruzi* | |
|---|---|---|---|---|---|---|---|---|---|---|
| Country | Cov.[d] (%) | Prev.[e] (/10³) | Cov. (%) | Prev. (/10³) | Cov. (%) | Prev. (/10³) | Cov. (%) | Prev. (/10³) | Cov. (%) | Prev. (/10³) |
| Bolivia | 36.2 | 0.10 | 14.5 | 2.00 | 0 | ?[f] | 37.9 | 18.10 | 29.4 | 147.90 |
| Chile | 100 | 3.40 | 98.7 | 2.00 | 34.0 | 6.40 | 95.2 | 11.40 | 76.7 | 12.00 |
| Colombia | 98.8 | 2.00 | 98.3 | 7.00 | 24.7 | 9.00 | 87.3 | 13.00 | 1.4 | 12.00 |
| Costa Rica | 100 | 0.34 | 100 | 4.50 | 0 | ? | 100 | 5.00 | 0 | ? |
| Ecuador | 89.5 | 1.00 | 88.2 | 3.80 | 32.9 | 1.40 | 86.7 | 11.50 | 51.0 | 2.00 |
| El Salvador | 100 | 1.30 | 96.0 | 8.00 | 31.4 | 2.50 | 100 | 19.00 | 42.5 | 14.70 |
| Guatemala | 100 | 3.00 | 79.8 | 7.00 | 37.2 | 8.00 | 100 | 19.00 | 75.0 | 14.00 |
| Honduras | 100 | 3.90 | 83.5 | 2.70 | 27.8 | 0.50 | 100 | 7.00 | 100 | 12.40 |
| Nicaragua | 100 | 0.04 | 53.1 | 4.00 | 53.1 | 4.40 | 88.4 | 16.00 | 58.4 | 2.40 |
| Paraguay | 100 | 0.70 | 92.9 | 13.00 | 0 | ? | 66.9 | 28.00 | 86.8 | 45.00 |
| Peru | 100 | 2.80 | 100 | 8.60 | 57.4 | 4.40 | 100 | 9.60 | 0 | 23.60[g] |
| Venezuela | 100 | 2.10 | 100 | 14.40 | 31.0 | 9.40 | 100 | 10.70 | 100 | 13.20 |

[a]Coverage of screening = (number of screened donors ÷ total number of donors) x 100.
[b]Data as reported by the countries from 1993, except for Ecuador and Paraguay, which were for 1994.
[c]HBsAg only.
[d]Coverage.
[e]Prevalence.
[f]Screening not performed and prevalence not known.
[g]Data from a survey of 2,237 samples.

*P(R)* multiplied by the infectivity risk. For countries reporting 100% of screening coverage for a specific disease, a residual *P(R)* was estimated as prevalence x 1-screening sensitivity (Table 2). Infectivity risk (defined as the likelihood of being infected when receiving an infected transfusion unit) was assumed to be 90% for HIV (13), 75% for HBV (14), 90% for HCV (15), and 20% for *T. cruzi* (16) (Table 2). Estimates for transfusion-acquired syphilis are not presented because the infectivity risk depends on length of refrigeration (17).

Considering the low prevalence rates and the incompleteness of HIV screening, only Bolivia, Colombia, and Ecuador could have missed detecting an HIV-infected transfusion unit; the probability of getting an infection in these countries was estimated at 0.57, 0.22, and 0.95 per 10,000 transfusions, respectively. For HBV and HCV this risk is higher. Up to 14.21 HBV infections (Nicaragua) and 67.09 HCV infections (Colombia) per 10,000 transfusions may have occurred. The highest risk for transfusion-transmitted infection was estimated for *T. cruzi:* 219.28 per 10,000 and 49.56 per 10,000 for Bolivia and Peru, respectively, and approximately 2 to 24 per 10,000 for the other seven countries (Table 2).

Table 3 shows estimates of the absolute number of infections that may have been induced

Table 2. Probability of receiving an infected transfusion *P(R)*[a] and probability of getting a transfusion-transmitted infection *P(I)*[b], by country[c]

| | HIV (x10⁴) | | HBV (x10⁴) | | HCV (x10⁴) | | *T. cruzi* (x10⁴) | |
|---|---|---|---|---|---|---|---|---|
| Country | *P(R)* | *P(I)* | *P(R)* | *P(I)* | *P(R)* | *P(I)* | *P(R)* | *P(I)* |
| Bolivia | 0.64 | 0.57 | 17.27 | 12.95 | NSP[d] | NSP | 1096.38 | 219.28 |
| Chile | 0.00 | 0.00 | 0.26 | 0.20 | 46.46 | 41.82 | 29.36 | 5.87 |
| Colombia | 0.24 | 0.22 | 1.20 | 0.90 | 74.55 | 67.09 | 124.24 | 24.85 |
| Costa Rica | 0.00 | 0.00 | 0.45[x] | 0.34 | NSP | NSP | NSP | NSP |
| Ecuador | 1.05 | 0.95 | 4.52 | 3.39 | 10.33 | 9.38 | 10.29 | 2.06 |
| El Salvador | 0.00 | 0.00 | 3.23 | 2.42 | 18.87 | 16.97 | 88.75 | 17.75 |
| Guatemala | 0.00 | 0.00 | 14.28 | 10.71 | 55.26 | 49.74 | 36.75 | 7.35 |
| Honduras | 0.00 | 0.00 | 4.49 | 3.37 | 3.97 | 3.57 | 13.02[x] | 2.60 |
| Nicaragua | 0.00 | 0.00 | 18.95 | 14.21 | 22.70 | 20.43 | 10.48 | 2.10 |
| Paraguay | 0.00 | 0.00 | 9.32 | 6.99 | NSP | NSP | 62.37 | 12.47 |
| Peru | 0.00 | 0.00 | 0.87[x] | 0.65 | 20.62 | 18.56 | 247.80 | 49.56 |
| Venezuela | 0.00 | 0.00 | 1.45[x] | 1.09 | 71.35 | 64.21 | 13.86[x] | 2.77 |

[a]*P(R)* = probability of receiving an infected transfusion = prevalence of infection x 1- level of screening; [x]for countries in which reported screening level was 100%, a residual *P(R)* was estimated as prevalence x 1- screening sensitivity rate x 10,000.
[b]*P(I)* = probability of getting a transfusion-transmitted infection = *P(R)* x infectivity index (infectivity indexes used were HIV=90%; HBV=75%; HCV=90%; *T.cruzi*=20%). For calculations of *P(R)* and *P(I)* the prevalence was corrected taking into account the sensitivity of the screening.
[c]Data from 1993, except for Ecuador and Paraguay, which were for 1994.
[d]No Screening performed, so *P(R)* and *P(I)* not known.

by transfusion, calculated as [no. of donors x *P(I)*], for each country. Because Chile (6) and Peru (3) reported fractionation of blood units by 1.85 and 1.5, respectively, the estimated number of infected units transfused in those countries was multiplied by these factors. For the remaining countries, it was assumed that each donated unit was given to only one recipient. An index of infectious disease spread through blood transfusion was calculated by dividing the estimated total number of transfusion-related infections (for any one of the infectious agents considered) by the total number of donors. This index indicates the health risks associated with blood transfusion and can be used as an outcome indicator to assess the cost-effectiveness of screening programs.

The highest value for the infection spreading index was obtained for Bolivia, where 233 transfusion-related infections may have occurred per 10,000 donations. This was a result of a very high prevalence rate of antibodies to *T. cruzi* and a lower level of screening. For most other countries considered, the index was 68 to 103 infections per 10,000 donations. Due to low seroprevalence rates and good screening levels in some cases, the risk for transfusion-related infections was relatively low in Honduras (nine per 10,000), Ecuador (16 per 10,000), and Paraguay (19 per 10,000).

Table 3 also shows the ratio of number of infections per donation by country. One infection (HIV, HBV, HCV, or *T. cruzi*) might have been transmitted in every 43 (Bolivia) to 1,072 (Honduras) donated units.

### Screening Costs

The unitary cost for serologic screening, estimated solely from expenditures on the least expensive laboratory reagents in each country and considering the prevalence rates reported by the countries was US$0.9 to US$2.4 for an HIV enzyme-linked immunosorbent assay (ELISA), US$0.5 to US$3.5 for HBV screening (enzyme immunoassay, radioimmunoassay, or passive reverse hemagglutination), US$3.5 to US$10.0 for HCV ELISA, US$0.25 to US$1.0 for a *T. cruzi* test (ELISA, radioimmunoassay, or indirect hemagglutination) (Table 4), and US$0.09 to US$0.60 for syphilis serology (RPR or VDRL). Using other tests might have increased the costs significantly for some of the infections. For example, the rapid agglutination test for HIV is usually more expensive than ELISA.

The cost of preventing the transfusion of one infected unit was estimated as [(no. of donors x cost of each test)/ total number of positive donors] as reported by each country. This value represents the cost of detecting one unit positive for any one of the infections studied in each country by using one diagnostic test for each infectious disease. For example, using two tests, one for antibody detection and one for antigen detection of HIV, increases costs. Detection of *T. cruzi* was the least expensive (US$11-$209 per positive unit), followed by HBV (US$90-USS$599 per unit), HCV (US$438-$7,136 per unit), and HIV (US$232-$23,000 per unit) (Table 4). The wide variation of cost primarily reflects differences in the prevalence of each infection and in the cost of each test in the countries.

The costs per capita to carry out a complete screening of blood donors in each country was US$0.008 to US$0.04 for HIV, US$0.008 to US$0.02 for

Table 3. Estimates of transfusion-transmitted infectious diseases, by country[a]

| Country | No. of donors | Absolute no. of transfusion-transmitted infectious diseases[b] HIV | HBV | HCV | *T. cruzi* | Total | Infection spreading index[c] /$10^4$ | Ratio of infections: donations |
|---|---|---|---|---|---|---|---|---|
| Bolivia | 37,948 | 2 | 49 | NA[e] | 832 | 883 | 233 | 1:43 |
| Chile[d] | 217,312 | 0 | 8 | 1681 | 236 | 1925 | 88 | 1:113 |
| Colombia | 352,316 | 8 | 32 | 2364 | 875 | 3279 | 93 | 1:107 |
| Costa Rica | 50,692 | 0 | 2[f] | NA | NA | NA | NA | NA |
| Ecuador | 98,473 | 9 | 33 | 92 | 20 | 154 | 16 | 1:639 |
| El Salvador | 48,048 | 0 | 12 | 82 | 85 | 179 | 37 | 1:268 |
| Guatemala | 45,426 | 0 | 49 | 226 | 33 | 308 | 68 | 1:147 |
| Honduras | 27,885 | 0 | 9 | 10 | 7[f] | 26 | 9 | 1:1072 |
| Nicaragua | 46,001 | 0 | 65 | 94 | 10 | 169 | 37 | 1:272 |
| Paraguay | 32,893 | 0 | 23 | NA | 41 | 64 | 19 | 1:514 |
| Peru[g] | 52,909 | 0 | 4[f] | 147 | 393 | 544 | 103 | 1:97 |
| Venezuela | 204,316 | 0 | 22[f] | 1312 | 57[f] | 1391 | 68 | 1:147 |

[a]Data from 1993 except for Ecuador and Paraguay, which were for 1994.
[b]Number of cases transmitted by blood transfusion = [number of donors x *P(I)* ]. For calculations of number of infections, the prevalence was corrected taking into account the sensitivity of the screening.
[c]Infection spreading index = (total number of infections transmitted ÷ number of donors) x 10,000.
[d]Fractionation index = 1.85.
[e]Data not available.
[f]Residual infectivity considering that sensitivity of diagnostic tests is not 100%.
[g]Fractionation index = 1.5.

Table 4. Estimated unitary cost of preventing transfusion-transmitted infections, by country, 1993[a]

| | Cost (US$) | | | | | | | |
|---|---|---|---|---|---|---|---|---|
| | HIV | | HBV[b] | | HCV | | *T. cruzi* | |
| Country | Single test | Preventing one infected unit | Single test | Preventing one infected unit | Single test | Preventing one infected unit | Single test | Preventing one infected unit |
| Chile | 2.3 | 676 | 1.2[c] | 599 | 3.5[d] | 547 | NA[e] | NA |
| Costa Rica | 1.1 | 3,280 | 0.5[c] | 111 | NA | NA | NA | NA |
| Ecuador[f] | 1.7 | 1,708 | 1.0[c] | 263 | 10.0 | 7,136 | 0.35[g] | 175 |
| El Salvador | 2.0 | 1,550 | 1.9[c] | 238 | 4.5 | 1,802 | 1.0[g,h] | 68 |
| | | | | | | | 1.0 | |
| Guatemala | 1.8 | 601 | 1.7[c] | 243 | 3.5 | 438 | 0.9[g] | 65 |
| Honduras | 0.9 | 232 | 0.9[c] | 334 | 3.5 | 6,971 | 0.45[h] | 36 |
| | | | 0.5[h] | 186 | | | | |
| Nicaragua | 1.0 | 23,000 | 0.5[h] | 125 | 3.5 | 797 | 0.5[h] | 209 |
| Peru (Lima) | 2.4 | 858 | 3.5[c] | 407 | 8.2 | 1,862 | 0.25[g] | 11 |
| | | | 2.4[i] | 279 | | | | |
| Venezuela | 1.3 | 619 | 1.3[c] | 90 | 4.5 | 479 | 0.5 | 38 |
| | | | | | | | 0.3[g] | 23 |

[a]Cost of preventing (=detecting) one infected unit was calculated as [(number of donors x test cost) ÷ (total number of positive donors detected)]. All costs refer to enzyme-linked immunosorbent assay, unless otherwise indicated.
[b]HBsAg only
[c]Enzyme immunoassay.
[d]Estimated cost based on cost of test in other countries.
[e]Data not available.
[f]Donors and prevalence for 1994, costs for 1993.
[g]Indirect hemagglutination.
[h]Radioimmunoassay.
[i]Passive reverse hemagglutination.

HBV, US$0.01 to US$0.08 for HCV, US$0.0008 to US$0.003 for syphilis, and US$0.0025 to US$0.009 for *T. cruzi*.

### Condition of the Blood Supply

These estimates indicate that the condition of the blood supply in Central and South America is far from ideal. Roughly, one case of transfusion-related infection occurs every 43 to 1,072 donations, varying with the infectious agent and the country.

In three of the 12 countries, transfusion recipients might become infected with HIV; in nine countries, with HCV; in all countries, with HBV; and in ten countries, with *T. cruzi*. However, it was not possible to establish the potential number of tainted units/infections from countries in which there was no information on donor prevalence for HCV (e.g., Bolivia, Costa Rica, and Paraguay).

No serologic tests for *T. cruzi* were done in Costa Rica and Peru. Data on the prevalence of *T. cruzi* serology in blood donors from Costa Rica from 1983 to 1985 (18,19) suggest a risk. Data from a recent report of a survey among donors in Lima indicate a prevalence of 2.36% (3). If this is the real prevalence in the city, the number of tainted units transfused would have been 1,872 in 1993, while the number of persons infected through blood transfusion could have been 393.

On the other hand, considering the number of donors and the prevalence of the infection in the 12 countries, if blood had not been screened at all, more than 35,000 infected units would have been transfused. However, infections have different patterns of evolution. HIV-infected persons are expected to get AIDS at some time during their lives (20), while only 50% and 38% of persons, respectively, will get posttransfusion hepatitis after infection with HBV or HCV (21). On the other hand, 20% to 30% of those infected with *T. cruzi* will get Chagas disease (16,18,19).

### Limitations of the Data

Difficulties and limitations of the use of public health data for policy decisions, even in industralized countries, are well recognized. Figures presented here were generated to establish an approximation of the problem by providing an overview of the risk of receiving tainted blood in different countries in Central and South America. One potential cause of underestimation of viral infections transmitted by blood is the residual risk because of the window period, even when 100% of donors are screened by serology (8,9). However, this residual risk would be difficult to ascertain in most countries of Central and South America. Investigation of clinically identified cases after a transfusion, follow-up of recipients for seroconversion, and special laboratory studies detecting seronegative donors for missed infections are laborious and expensive and could seldom be undertaken in those countries. Another possibility would be studies that combine estimates of incidence rates of infection among repeated and first-time donors (who seroconvert) with estimates for the duration of the preseroconversion period for a specific infectious agent (22). Excellent results were obtained by this method in the United States, where more than 80% of donations come from repeat donors. Those studies involved hundreds of thousands of donors and millions of donations. To have incidence data on repeat donors, it is necessary to have a significant number of voluntary donors who will repeat donations. Therefore, it is unlikely that studies of that sort could be carried out in the countries mentioned here. First, the population of the countries is much smaller; therefore, the number of donations is smaller. For example, in all Central America the number of donations is approximately 210,000 per year. Second, the number of repeat donations from voluntary donors is small. Voluntary donors accounted for 30% and 40% of all donations in Colombia and Costa Rica and 4% to 10% of all donors in Chile, Bolivia, Peru, and Venezuela (2-6). The number of voluntary donors was also small in the remaining countries. In all countries of Central and South America, most donations come from directed donors, relatives or friends of patients. In addition, there is no national registry of donors to allow for follow-up. Using incidence rates for first-time donors instead of repeat donors is not a solution because official incidence rates for HIV or other viruses were not available at the time of this study.

The risk for transfusion-related infection could also be overestimated. Recipients may already be infected. This is especially likely for *T. cruzi* infection in Bolivia, where the seroprevalence in the general population could be higher than 20% (18,19). Another source of overestimation is that only some of the cases detected by screening would be confirmed. In several countries, a confirmatory test is mandatory for HIV, syphilis, and HCV. However, as the primary function of blood banks is donor screening, seropositive donors for any of the diseases mentioned here are usually referred to specialized services or reference laboratories for confirmation of the results of the screening, and if results are confirmed, for treatment and counseling. Results of this confirmatory serology are not often sent back to the blood bank, even when privacy concerns allow for it. Chile was the only country that reported results of confirmatory tests for HIV: results indicated that only 9% of those found positive by the screening were confirmed positive (6). With *T. cruzi*, as there is no confirmatory test, it is assumed that a true positive is a unit that is positive on more than one test. By these criteria, a recent study in Brazil suggested that only one out of five donors positive for *T. cruzi* could be considered a true positive (23). Those facts, however, do not reduce the public health relevance of the problem presented here, although the real numbers of potentially infected units/infected persons may be still lower than our estimates.

Establishment of a screening process in every country will depend on balancing the benefits and costs. Although costs for preventing transfusion of one tainted unit or preventing one infection seem high for some etiologic agents, they are not so. Even in the case of Nicaragua, the country with the lowest HIV prevalence, the cost to prevent the transfusion of one potentially HIV-infected unit (by testing all donors with ELISA) was estimated at US$23,000, while treatment costs (drugs only) for an AIDS patient would be approximately US$12,000 per year.

In general, the risk for an infectious disease through tainted transfusion is not as high as that reported from some countries of Africa (24). Since 1993, donor screening has improved in several countries. Chile, Colombia, Costa Rica, and Venezuela, for example, have made screening for HCV mandatory, and coverage for serology for that infection has increased in those countries, as well as in El Salvador and Honduras. *T. cruzi* screening is now mandatory in Colombia, and the percentage of screened donors not only increased in Colombia but also in Ecuador, El Salvador, and Paraguay. Laws to regulate blood transfusion practices have been enacted in Bolivia, Guatemala, and Peru. The figures presented, however, underline the need for improvement and stress the importance of an information system that allows assessing the level of screening for infectious diseases in the blood supply. Universal screening of donors for HCV is still a priority in most countries, and increased donor screening for *T. cruzi* is a priority for Bolivia and possibly for Peru.

Continuous collection of the type of information shown here, which has only been partially available (1), provides a baseline against which future achievements can be measured and is essential for obtaining the support needed to maintain or expand the screening of blood donors.

### References

1. Linares J, Vinelli E, editors. Taller Latinoamericano de servicios de transfusión sanguínea y óptimo uso de los recursos. Cruz Roja Finlandesa; 1994. p. 167.
2. Organización Panamericana de la Salud. Taller para el control de calidad de sangre en transfusiones: serología para la detección de Chagas, hepatitis B y C, sífilis y HIV/SIDA. Document OPS/HPC/HCT/94.42.
3. Organización Panamericana de la Salud. Países andinos. Taller sobre control de calidad de sangre en transfusiones: serología para la detección de hepatitis B y C, sífilis, tripanosomiasis americana y VIH/SIDA. Document OPS/HCP/HCT/95-61.
4. Organización Panamericana de la Salud. Simposio internacional sobre control de calidad en bancos de sangre del Cono Sur y de Brasil. Informe final OPS/HCP/HCT/95.55.
5. Organización Panamericana de la Salud. Taller sobre control de calidad en serología de bancos de sangre. OPS/HCP/HCT/96/79.
6. Ministerio de Salud, Chile. Diagnóstico de la situación de los bancos de sangre y medicina transfusionál en Chile 1993. Santiago, Chile: La Ministerio; 1995. Ser Inf Téc No.14.
7. Pan American Health Organization. Health conditions in the Americas. Washington (DC): The Organization; 1994. PAHO Sci Pub No. 549.
8. Lackritz EM, Satten GA, Aberle-Grasse J, Dodd RY, Raimondi VP, Janssen RS, et al. Estimated risk of transmission of the human immunodeficiency virus by screened blood in the United States. N Eng J Med 1995;333:1721-5.
9. Schreiber GB, Busch MP, Kleinman SH, Korelitz JJ. The risk of transfusion-transmitted viral infections. N Eng J Med 1996;334:1685-90.
10. Van de Poel CL, Cuypers HT, Reesink HW. Hepatitis C virus six years on. Lancet 1994;344:1475-9.
11. Andrade ANS, Martelli CMT, Luquetti AO, Oliveira OS, Almeida e Silva S, Zicker F. Triagem sorologica ra o Trypanosoma cruzi entre doadores de sangue do Brasil central. Bol Oficina Sanit Panam 1992;113:19-27.
12. Lorca MH, Child RB, Garcia AC, Silva MG, Osorio JS, Atias M. Evaluación de reactivos comerciales empleados en el diagnóstico de la enfermedad de Chagas en bancos de sangre de Chile. I Selección de reactivos. Rev Med Chile. 1992;120:420-6.
13. Donegan E, Stuart M, Niland JC, Sacks HS, Azen SP, Dietrich SL, et al. Infection with human immunodeficiency virus type 1 (HIV-1) among recipients of antibody-positive blood donations. Ann Intern Med 1990;113:733-9.
14. Gocke DJ. A prospective study of post-transfusional hepatitis. JAMA 1972;219:1165-70.
15. Aach RD, Stevens CE, Hollinger FB, Mosley JW, Peterson DA, Taylor PE, et al. Hepatitis C virus infection in post-transfusion hepatitis. An analysis with first- and second-generation assays. N Engl J Med 1991;325:1325-9.
16. World Health Organization. The control of Chagas disease. Geneva: WHO Tech Rep Ser No.811:32;990.
17. National Institutes of Health. Infectious disease testing for blood transfusion. NIH Consens Statement 1995;13:13-4.
18. Schmunis GA. *Trypanosoma cruzi*, the etiologic agent of Chagas disease: status in the blood supply in endemic and non endemic countries. Transfusion 1991;31:547-55.
19. Schmunis GA. American trypanosomiasis as a public health problem. Chagas disease and the nervous system. PAHO Sci Pub 994;547:3-29.
20. Pedersen C, Lindhardt BO, Jensen BL, Lavritzen E, Gerstoft J, Dickmeiss E, et al. Clinical course of primary HIV infection: consequences for subsequent course of infection. BMJ 1989;299:154-7.
21. Alter M. Residual risk of transfusion associated hepatitis. In: Program and Abstracts of the National Institutes of Health Development Conference on Infectious Diseases Testing for Blood Transfusions. 1995 Jan 9-11; Bethesda (MD): National Institutes of Health;1995. p. 23-7.
22. Busch MP. Incidence of infectious disease markers in blood donors, implications for residual risk of viral transmission by transfusion. In: Program and Abstracts of the National Institutes of Health Development Conference; 1995. Jan 9-11; Bethesda (MD): National Institutes of Health; 1995. P. 29-30.
23. Hamerschlak N, Pasternak J, Amato Neto V, Carvalho MB, Guerra CS, Coscina AL, et al. Chagas' disease, an algorithm for donor screening and positive donor counseling. Rev Soc Bras Med Trop 1997;30:205-9.
24. McFarland W, Mvere D, Shandera W, Reingold A. Epidemiology and prevention of transfusion-associated human immunodeficiency virus transmission in sub-Saharan Africa. Vox Sang 1997;72:85-92.
